# Influence of Surface-Active Molecules in Solution on Charge Transfer Due to a Water–Air Contact Line Moving over a Hydrophobic Surface

**DOI:** 10.1021/acs.langmuir.5c00043

**Published:** 2025-04-14

**Authors:** L. E. Helseth

**Affiliations:** Department of Physics and Technology, University of Bergen, Allegaten 55, Bergen 5020, Norway

## Abstract

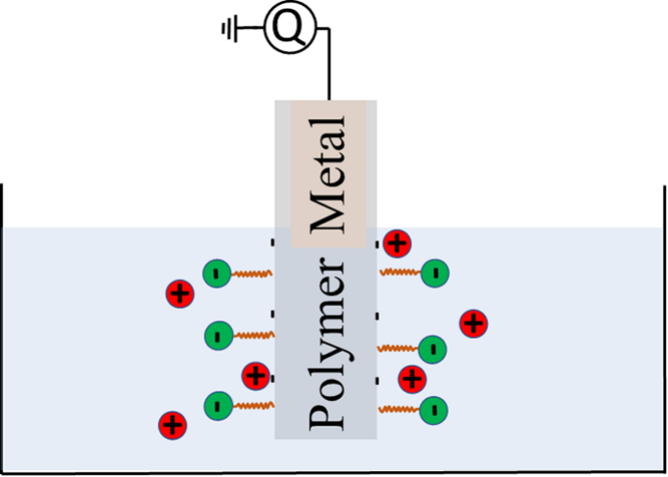

Charge transfer due to a water–air contact line moving over a fluoropolymer hydrophobic surface is investigated for an aqueous solution containing surface-active molecules. It is found that anionic (SDS) and neutral (Triton X-100) surfactants exhibit a two-stage charge transfer reduction with concentration. At low concentrations, a layer of surfactant molecules accumulates near the hydrophobic surface and partially quenches the charge transfer. Surprisingly, after this first stage, the charge transfer remains nearly constant or weakly increasing, while the concentration of surfactants increases several orders of magnitude. Eventually, for large enough concentrations, the charge transfer continues to decrease, eventually resulting in almost zero charge transfer before reaching the critical micelle concentration. For the cationic surfactant (CTAB), the behavior is entirely different and a single quenching mechanism can explain the observed reduction in charge transfer due to positively charged surface-active molecules forming a layer that electrostatically screens the water-induced negative charge residing on the hydrophobic interface. A similar behavior is observed for poly(vinyl alcohol), which is attributed to its known and strong interaction with the hydrophobic surface used in this study.

## 1 Introduction

Contact between a hydrophobic polymer surface and an aqueous solution leads to charge transfer.^1−7^ The charge transfer depends on a range of different parameters such as the surface roughness,^8^ triboelectric charge state,^9−15^ substrate chemistry,^16^ composition and ion strength of the aqueous solution,^17−23^ flow rate,^24^ and distance moved over the solid surface.^2,25^

The water–air contact line moving over a hydrophobic surface has been investigated by studying the movement of droplets,^26−33^ wave motion,^34,35^ air bubbles,^36−38^ or by dipping hydrophobic surfaces into solution.^20,39,40^ While the influence of pH,^21,41,42^ ions,^39,41^ miscible alcohols,^40^ or surfaces altered by amphiphilic compounds^42^ on charge transfer has been investigated both experimentally and theoretically, less work has been done to understand the impact of water-soluble surfactants. Surfactants soluble in aqueous solutions are known to influence the interfacial tension^43−46^ and interfacial properties of hydrophobic surfaces,^47−49^ depending on whether they are nonionic, anionic, or cationic. However, less is known how they alter charge transfer as the three-phase contact line passes over a hydrophobic surface and, in particular, whether this can advance our knowledge of how the surfactants interact with hydrophobic surfaces at different concentrations. This will allow not only a better understanding of the underlying mechanisms but also provide insight into how surfactants may influence the performance of energy harvesting mechanisms utilizing solid–liquid charge transfer.^26−31,34−38^ Surfactants are often present in aqueous solutions in very small concentrations and have the potential to significantly alter the charge transfer in a manner that should be better understood.

Here, these issues are addressed, and the influence of surface-active molecules on the charge transfer at a hydrophobic fluoropolymer surface is investigated. The charge transfer versus concentration is investigated for surfactants with nonionic, anionic, and cationic hydrophilic headgroups. The results are compared with the charge transfer in the presence of poly(vinyl alcohol), which is known to adsorb particularly well to fluoropolymer surfaces,^48,49^ in order to have a better understanding of the observed charge transfer quenching.

## 2 Materials and Methods

### 2.1 Materials

**Figure 1:**
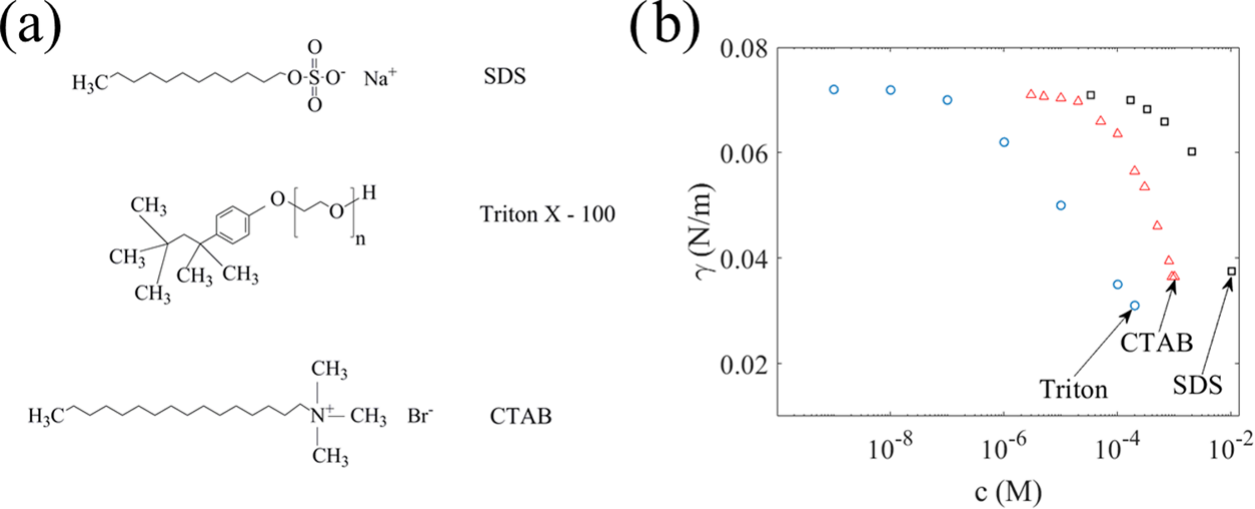
(a) Molecular structure and counterions of the surfactant molecules studied in this work. (b) Surface tension of SDS (black squares), CTAB (red triangles), and Triton X-100 (blue circles) at the air–water interface as a function of molar surfactant concentration. The data for CTAB and Triton X-100 are extracted from refs^45^ and^43^, respectively. The arrows indicate the critical micelle concentration (cmc) for each of the surfactants in water.

Sodium dodecyl sulfate (SDS), cetyltrimethylammonium bromide (CTAB), Triton X-100, and poly(vinyl alcohol) (PvOH, M.W. 1,30,000) were obtained from Sigma-Aldrich. The molecular structure of the three surfactants is shown in Figure 1a. Deionized, ultrapure water (18.2 MΩcm, Millipore) was used to make all of the solutions. It was found that consistent charge transfer measurements in open air were obtained if freshly produced deionized water was used and the measurements in open air were done within 2 h, thus suggesting that possible contamination from the environment (e.g., gases or ions) did not play a role under such circumstances.

### 2.2 Surface Tension

In Figure 1b, the surface tension of CTAB (red triangles) at the air–water interface was extracted from the data presented in ref (45) and presented as red triangles, whereas the surface tension of Triton X-100 at the air–water interface was extracted from ref (43) and presented as blue points. A self-made fiberoptic sensor of the type detailed in ref (50) with a 5-mm-wide and 45-mm-long Whatman No. 4 filter paper as a probe was lowered into water to which the surfactants were added. In this manner, the surface tension at the air–water interface was measured and found to be comparable to the literature values in refs^43−46^. The surface tension at the air–water interface decays monotonously with concentration, as shown for the extracted data for CTAB and Triton X-100 in Figure 1b. For CTAB, the critical micelle concentration (cmc) is about 1 mM, whereas for Triton X-100 it is 0.1 mM. For each of the surfactants, the surface tension at the air–water interface remained almost constant at concentrations higher than the respective cmc value; see also refs^43,45^. The black squares in Figure 1b show the surface tension of SDS at the air–water interface versus concentration measured with the self-made fiberoptic setup, and is also found to coincide with data found in the literature.^46^ The cmc is found at about 9 mM, and the surface tension remains constant above that value.

### 2.3 Dipping-Probe Charge Transfer Measurements

**Figure 2:**
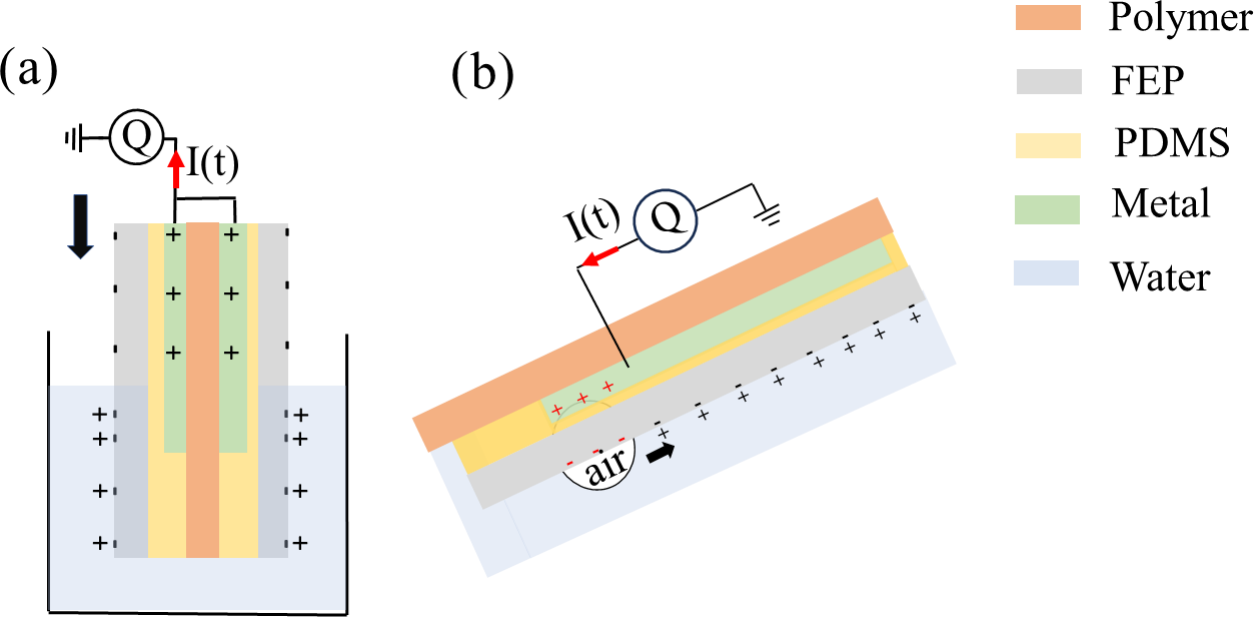
Experimental setups for dipping measurements (a) and air-bubble measurements (b) of charge transfer. The brown-colored polymers in panels (a) and (b) are either polystyrene or polyacrylate, used as a backbone to mount the FEP and metal electrodes on. In panel a, the FEP-covered metal electrode is dipped into the water, whereas in panel b, an air bubble moves across the FEP-covered metal electrode. In both cases, the resulting current is shown with a red arrow, and the corresponding charge was recorded by using an electrometer. See the text for details.

The charge transfer is investigated using the same setup and dipping technique described in detail in refs^20,39,40^. A schematic drawing of the setup is shown in Figure 1a. To make the dipping probe, a 2-mm-thick polystyrene piece was cut to be 50 mm tall and 22 mm wide and covered partially by a 0.03-mm-thick aluminum tape. Polystyrene and metal were then covered by fluorinated ethylene propylene (FEP) with a thickness of 50 μm (Dupont). To ensure adhesion of the fluoropolymer film and to avoid water contacting the metal, polydimethylsiloxane (PDMS) was used as glue. The dipping probe was mounted on an electromagnetic shaker (Smart Materials GmbH) such that the probe could be dipped up and down at a selected frequency and amplitude. Here, we used an oscillation amplitude of 8 mm at a frequency of 2.3–2.4 Hz, corresponding to a velocity of about 0.1 m/s, as also used in refs^39,40^. The charge measured as the probe was dipped into, removed from the solution, and was measured using a Keithley 6514 electrometer, marked by a “Q” in Figure 2.

**Figure 3:**
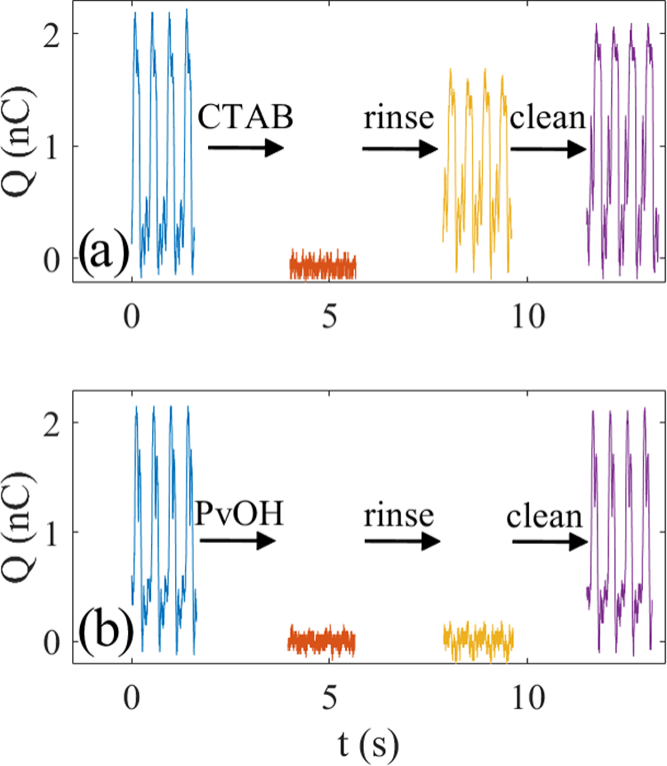
Application, rinsing, and cleaning procedure is shown when 0.5 μM CTAB (a) and 0.2 mg/L PvOH (b) are added to deionized water. In both panels (a) and (b), the deionized water exhibits a maximum charge transfer of about 2 nC and is represented by blue lines in the left of the graphs, whereas the deionized water after cleaning is depicted as violet lines on the right.

A typical procedure showing the process used to measure charge transfer as a function of surfactant concentration is shown in Figure 3a. First, the FEP-covered metal electrode is cleaned thoroughly by wiping it with a clean paper containing methanol, and thereafter rinsing it in methanol 2–3 times before flushing it with copious amounts of deionized water. The probe is then inserted in deionized water and dipped up and down using the described vibration mechanism, resulting in the measured charge *Q* versus time shown as the leftmost, blue curve in Figure 3a. The charge transfer represents the maximum peaks in Figure 3a, which is hereafter called Δ*Q*_m_.

If the charge transfer measured is 2.0 ± 0.2 nC, then the measurements can continue. If not, then an additional cleaning procedure is needed. When this calibration is complete, a solution containing the surface-active molecules is added. The red curve in Figure 3a shows that when about 0.5 μM CTAB is used, the measured charge transfer is very small. If the probe is rinsed once in deionized water, the measured charge transfer almost reaches that measured in pure deionized water on a properly cleaned probe. However, it was found that in order to measure a charge transfer within the accepted calibration range of 2.0 ± 0.2 nC, thorough cleaning with methanol and water as described above was necessary. After such a clean, the measured charge transfer in deionized water (violet curve on the right) would go back to approximately the same as it was initially (blue curve on the left; see Figure 3a, within the uncertainty range given).

A similar procedure was applied also to SDS and Triton X-100, both of which showed similar behavior as CTAB. That is, after a certain concentration, the charge transfer was mostly quenched, but by rinsing in deionized water, one could regain most of the charge transfer. However, a thorough clean as described above was needed to fully regain the charge within the range 2.0 ± 0.2 nC. PvOH stood out among the investigated substances, as demonstrated in Figure 3b. After adding a water-based solution of PvOH resulting in 0.2 mg/L (corresponding to 1.5 nM for a molecular weight of 1,30,000) dissolved in deionized water, the charge transfer measured was very small as shown in the red curve in Figure 3b. Rinsing the dipping probe with deionized water did not appear to improve the charge transfer significantly, as shown in the yellow curve in Figure 3b. That is, almost no change in the measured charge was found when the probe was dipped in a new volume of deionized water. Even after flushing the probe with copious amounts of water, the charge measured did not increase significantly. However, a thorough clean with methanol and water as described above would remove the remaining PvOH molecules, and the probe would once more generate a charge transfer within the accepted range in deionized water, as shown for the violet curve on the right in Figure 3b.

### 2.4 Air-Bubble Charge Transfer Measurements

**Figure 4:**
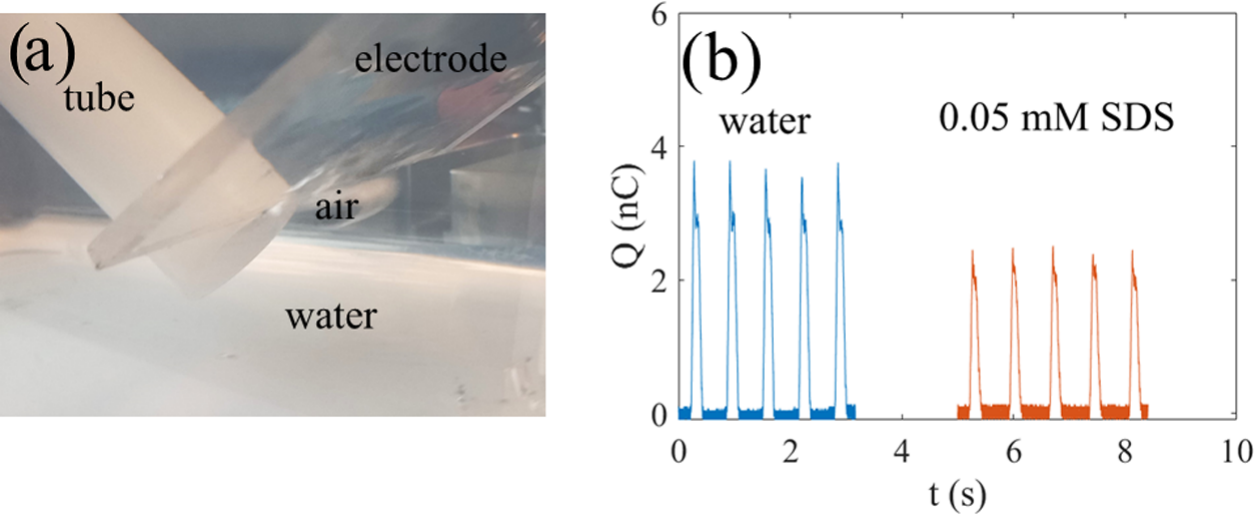
(a) Experimental setup for air-bubble measurements. (b) Measured charge versus time for deionized water and 0.05 mM SDS as five air bubbles pass by the edge of the FEP-covered metal electrode.

In order to evaluate the influence of surfactants on air-bubble energy harvesting, as described in e.g., refs^36−38^, it was decided to measure the charge transfer versus surfactant concentration using such a device. A device as described in ref (38) was used, with a schematic drawing of the setup shown in Figure 2b and an actual picture of the setup in Figure 4a. This setup also utilizes an aluminum film as the metal electrode. The metal was glued with PDMS to an acrylic plate and then covered with an FEP film of 50 μm thickness such that no liquid came into contact with the metal. The FEP film was positioned under water at an angle 40° with the water surface, and a pump provided a supply of air bubbles, which had a volume of about 2 mL moving at about 0.2 m/s. The bubbles moved upward due to buoyancy and had an elliptical shape of 3 cm width perpendicular to the motion and 1.5 cm tall in the direction of motion. Both the metal and the FEP were much wider than the air bubbles, such that edge effects did not influence the collected charge. For smaller bubbles, the three-phase contact line between air and water was found to exhibit a static contact angle θ = 70° if the FEP surface was positioned horizontally. For the large bubbles used in this study, the static contact angle was slightly larger, and the receding and advancing contact angles quickly fluctuated as the droplet moved across the tilted FEP.

In Figure 4b, the measured charge as a function of time for deionized water and 0.05 mM SDS is shown. In both the cases, the curve shapes are similar and the difference between the top and bottom is extracted and called the charge transfer. The length of the portion of the metal electrode covered by FEP under water is about 5 cm, and it was found that increasing this length did not influence the measured charge transfer. In each of the curves in Figure 4b, five air bubbles were passed over the position of the edge of the metal electrode, resulting in five charge pulses, which each last about 0.2 s. The curve shapes are different from those obtained using the dipping measurements detailed in the previous section, since the charge pulse has a duration that depends on the contact time with the FEP-covered metal electrode. Since the velocity of the air bubbles is about 0.2 m/s, the distance covered is about 4 cm during a pulse, i.e., comparable to the length of the metal electrode covered by FEP under water. A small discrepancy could be due to fluctuations or bubble liftoff from the FEP surface upon approaching the air–water interface, although such effects cannot be identified from Figure 4b.

During the measurements, it was found that the charge transfer could be measured with an uncertainty of about 10%, amounting to 3.5 ± 0.4 nC for pure water. However, it was also found that coupling the air bubble to the FEP was important to ensure reproducibility between experiments. This was done by aligning the tube where the air came from the polymer surface over which the air bubble was gliding, as shown in Figure 4a. In this manner, the air bubble may form an air volume in direct contact with the FEP as shown schematically in Figure 2b. If the tube was fixed too far from or not aligned with the FEP surface, the air bubbles would bounce such that air would only contact the FEP surface briefly, and the charge transfer would be strongly reduced. During the motion over the FEP surface, the air bubbles tended to fluctuate, and optical inspection could partially reveal how well the actual coupling to the FEP surface is or whether there is a very thin film of water between the air and the FEP. The uncertainty in charge transfer was relatively small, as reported above, which suggests that the procedure used is reproducible.

In both the dipping measurements discussed in Section 2.3 and the air-bubble measurements discussed in this section, surfactant-like molecules may, in principle, leak from the polymers in contact with water, in particular from PDMS used as glue. However, in both situations, the probes were carefully rinsed, cleaned, and tested before use. When the measurements were done in deionized water, no detectable changes in the measured charge transfer occurred when the system was left alone in operation for 1–2 h, which suggested that any leakage from the materials used to construct the probes was minimal and that contamination was not a problem.

The setup in Figure 4a was tested on different surfactants used in this study. However, in the case of Triton X-100, it was found that the bubbles generated built up on each other in a stable manner that disturbed the measurements. This bubble-build-up was not a significant problem with SDS and CTAB, and therefore measurements of only these two latter surfactants were undertaken with this setup.

## 3 Results and Discussion

### 3.1 Charge Transfer Due to SDS

**Figure 5:**
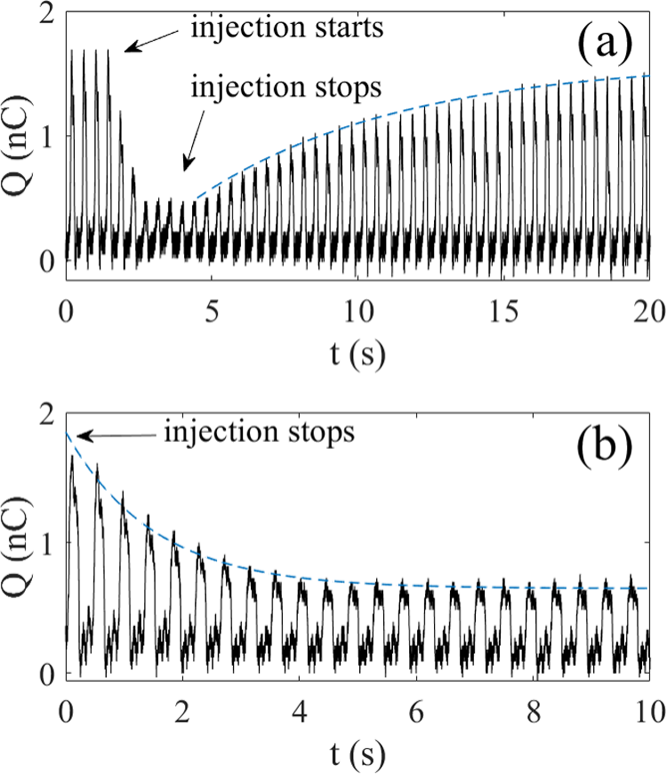
In panel (a), the charge transfer is deliberately quenched with excess SDS molecules by increasing the SDS concentration from 37 to 51 μM by adding 0.2 mL of 5 mM SDS. The blue, dashed line is a trend-line suggesting that the charge recovery follows first-order kinetics. In panel (b), the charge transfer is quenched by adding 1 μM CTAB to water, and the blue dashed trend-line suggests a first-order exponential decay.

The measurements of charge transfer were done after an incremental increase in concentration of surfactant at a rate less than 0.2 mL/s. It was found that the injection of large local concentrations of SDS temporarily quenched the charge transfer if the injection volumes are small enough. Figure 5a shows an example of this using the dipping probe of Figure 2a with an SDS concentration increasing from 37 to 51 μM by adding 0.2 mL of 5 mM SDS. This results in a temporary reduction in charge transfer from 1.8 nC to about *Q*_a_ = 0.5 nC. Here, the relatively large concentration is injected on purpose as close as possible to the dipping probe in the time interval between *t* = 1.7 and *t* = 3.8 s. Note that the charge transfer suppression kinetics observed between *t* = 1.7 and *t* = 3.8 s only depends on the way in which the SDS is injected. However, the subsequent kinetics is more interesting as the charge transfer gradually increases to about 1.4 nC starting at *t* ≈ 4 s. First-order kinetics has been used to model changes in wetting properties^51−53^ and is here utilized to model the gradual increase in charge transfer (i.e., the peaks) as Δ*Q*_m_=*Q*_a_+*Q*_b_[1–exp(−*t*/*t*_SDS_)], where the blue dashed line is a fit of this function using *Q*_b_ = 1.1 nC and *t*_SDS_ = 7.0 s. This time scale is within the range reported for adsorption of surfactants using surface tension measurements.^52^

In general, it was observed that even though the FEP surface is exposed to a large excess of SDS that quenches the charge transfer much beyond the total SDS concentration of the container, there will be a partial recovery of charge transfer. We speculate that this charge transfer recovery is due to a combination of diffusion and the shear force generated during dipping, removing the surfactant from the FEP surface and into the bulk in just a few seconds. Eventually, the ions near the surface and in the rest of the volume find a quasi-equilibrium state such that the charge transfer reaches a constant value. This mechanism only works at preexisting lower concentrations as shown in Figure 5a, since for higher SDS concentrations there are no available charge sites to quench and charge transfer recovery is not observed experimentally.

**Figure 6:**
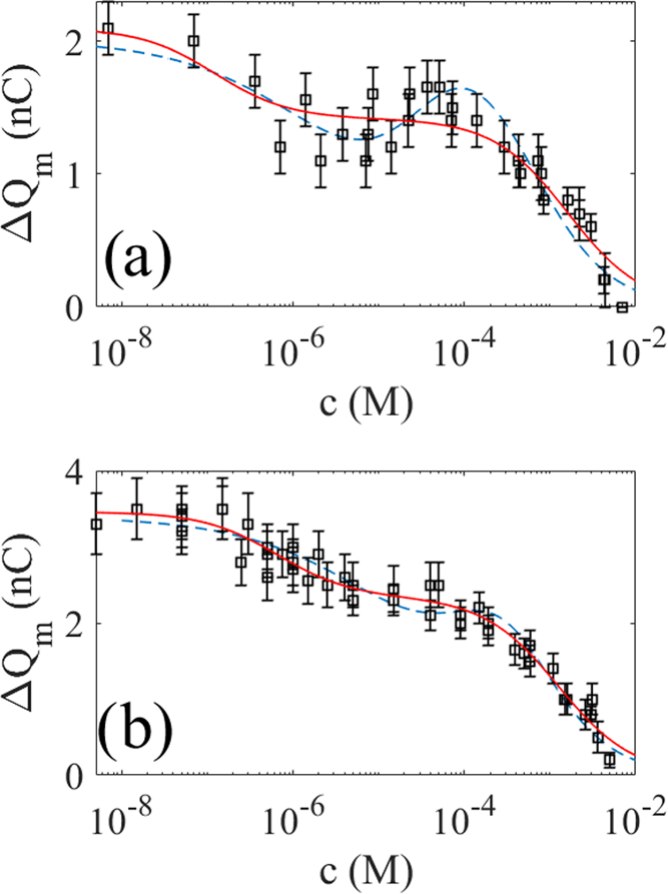
In panel (a), the squares are measured charge transfer versus SDS concentration using the dipping probe in Figure 2a, whereas in panel, (b) the squares represent the measured charge transfer using the air-bubble method in Figure 2b. The solid red lines are fits of eq 1, whereas the dashed blue lines are fits of eq 2 to the experimental data.

Subsequent measurements at different SDS concentrations were undertaken when the charge transfer had reached a stable value. The squares in Figure 6a show the measured charge transfer Δ*Q*_m_ using the dipping probe described in Figure 2a and Section 2.3. It is observed that for pure water the charge measured is about 2 nC, but that reduces gradually to 1.1–1.5 nC at 1 μM SDS. Increasing the SDS concentration further appears to increase the measured charge slightly until it reaches about 1.6–1.7 nC at 50 μM, after which it decreases monotonously to zero at about 10 mM SDS.

The squares in Figure 6b show the measured charge transfer using air bubbles as described in Figure 2b and Section 2.4. For deionized water, the charge transfer measured was about 3.5 nC, which is higher than that of the dipping probe 2 nC. The reason for this is that the air bubbles have an effective width of about 3 cm, whereas the dipping probe had a width of about 2.2 cm, which gives rise to a small relative increase in charge transfer due to the wider advancing front of the three-phase contact line. When the concentration of SDS increases in Figure 6b, one observes the same trend as for the dipping probe in Figure 6a, with a behavior that suggests a two-stage reduction of charge transfer. That is, the charge transfer is nearly constant at very small concentrations and then decreases monotonously until the concentration is about 1 μM, followed by a nearly constant charge transfer until 0.1 mM, after which it again decreases monotonously to zero at about 10 mM SDS.

The simplest model for the interpretation of the experimental data is a two-stage model exhibiting two separate and independent quenching mechanisms. We build the model on the framework of equilibrium theory for quenching of water activity proposed in ref (41) and used it in ref (40) to study the quenching of charge transfer due to mixtures of water with glycerol and methanol and also in ref (42) to study the charge transfer due to water droplets passing surface-modified polyethylene.

**Figure 7:**
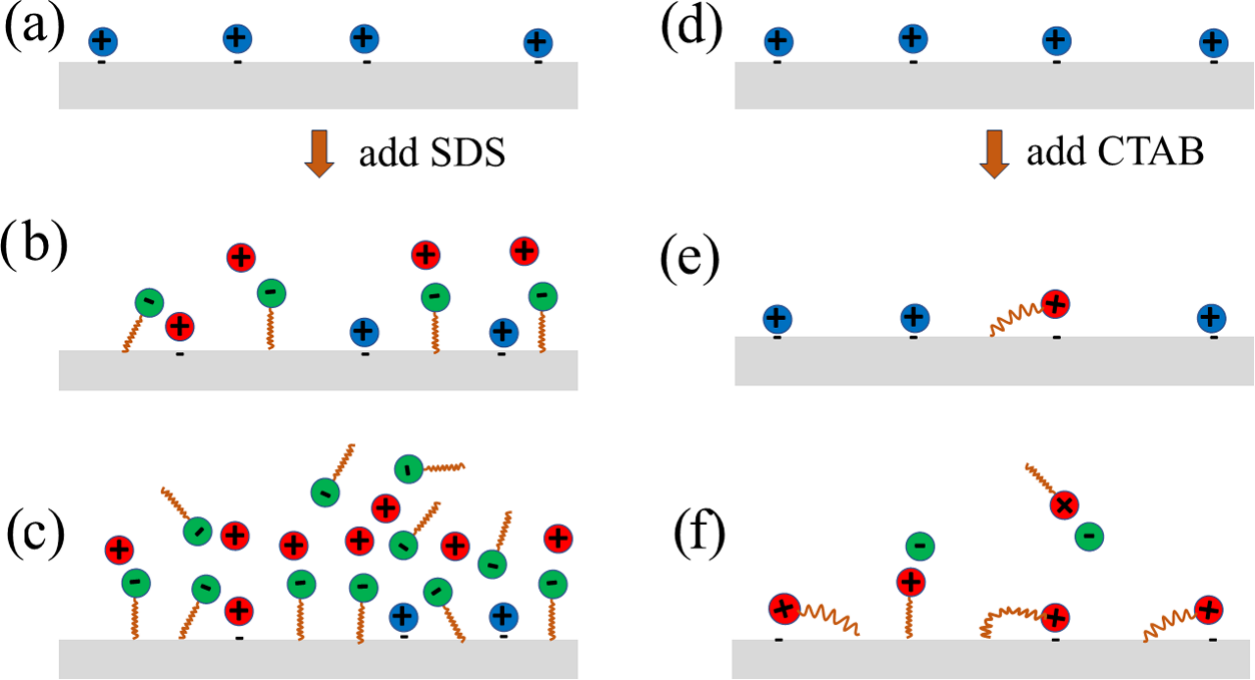
Artistic and schematic drawing of the charge transfer quenching by SDS and CTAB. Here, panel (a) shows the negative charge and surface protons formed by the hydrogen bond network at the FEP surface in pure water. In panel (b), SDS concentrations in the range of 1–50 μM are added, whereas in panel (c), the charge transfer is quenched at concentrations in the mM range before the formation of micelles. In panel (d), pure water is shown, in panel (e), a very small concentration of CTAB is added, while in panel (f), the charge transfer is fully quenched at CTAB concentrations of the order of 1 μM.

In pure water, the hydrogen bonding network is rearranged near the FEP surface, resulting in adsorption of negative charge (e.g., hydroxyl ions) and surface protons. The charge transfer is a result of the surface proton activity and the ease at which surface protons participate in the electrical double layer and can be removed as the three-phase contact line moves over the FEP surface, as discussed in ref (39). See Figure 7a for a simple artistic impression. The blue positive charges are the surface protons formed by the hydrogen network, located in the vicinity of the negative charge that forms at the FEP surface. When the three-phase contact line moves along the FEP surface, the positive charge in the diffuse part of the electrical double layer is removed by shear forces, and the negative charge remains on the surface.

When added to water, SDS dissociates into SDS^–^ and Na^+^ ions. SDS^–^ ions are active at the hydrophobic surface but may tend to locate with their hydrophobic tails at patches on the surface where there is little negative charge. This disrupts the hydrogen bond network, reduces the water activity, and either partially removes or blocks the surface protons and the negative charge already present. In other locations where this reduction of water activity does not take place, the positive sodium ions may interact with the negative charge on the FEP surface and contribute to the electrical double layer there. The red, positive ions in Figure 7b are Na^+^ ions. As pointed out in ref (39), small amounts of sodium ions should actually increase the charge transfer. The fact that this does not occur for very small concentrations of SDS suggests that the reduction in water activity due to SDS^–^ dominates. The SDS^–^ ions may also interact electrostatically with these sodium ions near the FEP surface in such a way as to quench at least some of the expected additional charge transfer due to sodium ions. Further studies of the molecular arrangement near the FEP would be needed to get a complete picture, but this is outside the scope and techniques available for the current work.

Upon the addition of SDS, some resilient negative surface charges remain. However, when enough surfactant anions are present at the FEP surface, a patchy monolayer is formed surrounding the resilient negative surface charges, and the first quenching stage is complete as shown in Figure 7b. Increasing the SDS concentration further does not change the measured charge transfer significantly, since most cations and surfactant anions coming from the bulk relocate to a position farther from the FEP surface, in the vicinity of the hydrophobic heads of the patchy monolayer, where they are removed together by the three-phase contact line without contributing to charge transfer. Thus, the charge transfer does not change significantly between 1 and 50 μM. It should however be noted that the addition of sodium cations may be the cause of the small increase in charge transfer seen in Figure 6a, as will be discussed in more detail below.

At higher concentrations of SDS, the surface protons and negative FEP surface charge are entirely screened by the large number of surfactant molecules surrounding them, as illustrated in Figure 7c. In this situation, no net positive charge can be transferred into water as the three-phase contact line moves over the FEP surface since both SDS^–^ ions and Na^+^ will be removed simultaneously and therefore retain electroneutrality. We hypothesize that when the three-phase contact line moves over the FEP surface, the patchy monolayer and secondary layer of SDS molecules mechanically shield the surface protons from being removed from the FEP surface. Moreover, the removed surfactants are quickly replaced, thus maintaining the situation seen in Figure 7c. The measurements presented in the current work cannot determine whether the original negative charge at the FEP surface has been entirely removed or is blocked or if it is a combination of these. However, the charge recovery measurements of Figure 5a may suggest that if one introduces high local concentrations of SDS^–^ and Na+ in an otherwise dilute solution, the ions will within seconds move away from the FEP surface such that charge transfer is recovered. Thus, the charge recovery observed in Figure 5a can be represented by a transition from the situation in Figure 7c and almost back to that in Figure 7b.

It should be emphasized that the charge is nearly entirely quenched at a concentration of about 10 mM, at which micelles are forming. Singular measurements taken above cmc indicate no measurable charge transfer, but systematic studies at such high concentrations are outside the scope of the current work. It is currently believed that the formation of micelles mainly takes place in the bulk since the FEP surface at these concentrations is rather crowded by surfactants. Thus, there is no reason to believe that micelles contribute to changes in the charge transfer.

As demonstrated in Figure 1b, the surface tension changes with the surfactant concentration, and one must therefore question whether the surface tension needs to be accounted for when modeling the system. The measurement setup used here as well as in the literature^46^ is not sensitive enough to find any changes in surface tension at the air–water interface at concentrations below 10 μM. However, estimates suggest that above 0.1 mM, the surface tension decreases by approximately 6 mN/m per 0.7 mM, which according to the Gibbs adsorption isotherm gives a surface excess of the order of 1 μM/m^2^ at the air–water interface, in reasonable agreement with the literature.^54^ Thus, at the air–water interface, the ions from SDS should in the higher concentration range occupy an area of about 2 nm^2^. The water–FEP interface differs from the air–water interface since the elastic interactions between the hydrophobic tail of the SDS^–^ and the solid interface must also be accounted for. At this point, the role of these elastic interactions is not clear but is hypothesized not to alter the area occupance significantly. If this is the case, it is useful to know how many molecules are required to alter the surface tension at the air–water interface as compared to the number of molecules required to alter the charge transfer at the water–FEP interface. The reduction in charge transfer is on the order of 1 nC, which corresponds to 10^–14^ mol of ions. If the dipping probe collects charge over an area 10^–5^ m^2^ as suggested in ref (39), the surface concentration is expected to be of the order of 1 nM/m^2^. This is much smaller than the surface excess found by using the Gibbs adsorption isotherm and the surface tension data. It should be pointed out that the probe does not collect all of the charge that is not screened, and as such it is not unexpected to get a smaller number from the charge transfer measurements than the surface tension measurements. However, the collected charge obtained from measurements is not expected to be 3 orders of magnitude smaller than the actual available charge, and one may conclude that the surface excess needed to reduce the charge transfer is much smaller than that needed to reduce the surface tension. For these reasons, it should be possible to build a model without explicitly including the surface tension at the water–FEP interface.

When SDS is introduced in water and SDS^–^ anions adsorb at the FEP surface, the local hydrogen bonding network is disrupted, and the transfer of charge is diminished. At small SDS concentrations (c), until the patchy monolayer is fully formed, the number of blocked charges in this patchy monolayer is *N*_b_, whereas the number of free charges is *N*_f_. The sum, *N*_b_ + *N*_f_, is assumed to be constant, and the fraction of free charge is γ_f_ = *N*_f_/(*N*_f_+*N*_b_) = 1/(1+*N*_b_/*N*_f_). If *q*_1_ is the magnitude of the charge that the patchy monolayer could quench, then the actual fraction of free charge is γ_f_*q*_1_. We assume now that *K*_1_c = *N*_b_/*N*_f_, where *K*_1_ is the equilibrium quenching constant, is the first quenching stage. The first stage, see Figure 7b, occurring at concentrations up until the patchy monolayer is fully developed and therefore gives rise to a charge transfer *q*_1_/(1+*K*_1_c).

If the concentration of SDS molecules becomes sufficiently large, then the number of surfactant anions will be large enough to remove or block the negative FEP surface charge entirely by populating the diffuse part of the electrical double layer outside the initial patchy monolayer of SDS^–^ formed during the first quenching stage discussed above. This can be modeled as a second stage of quenching; see Figure 7c. The simplest model is to assume that the quenching occurring in this second stage is independent of the first stage, thereby also assuming that the surfactant anions do not rearrange significantly after the patchy monolayer is formed. The charge transfer of the second stage is therefore associated with its own blocking phenomenon, wherein a charge *q*_2_ can be blocked by the surfactant anions. Using the same arguments as those for the first stage discussed above, one finds that the charge transfer is *q*_2_/(1+*K*_2_c), where *K*_2_ is the equilibrium quenching constant for the second stage. Here, only quenching due to surfactant anions is considered, but in principle, one should also account for quenching by sodium ions. As shown in ref (39), such quenching occurs only at higher concentrations of sodium ions, at which the quenching of surfactant anions already dominates, and is therefore neglected. In such a simple situation, the charge transfer is governed by two independent stages of quenching and the total charge transfer must be written as a sum
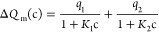
1where it should be noted that the charge in the absence of surfactant is *Q*_0p_ = *q*_1_ + *q*_2_, and that eq 1 only considers charge quenching by the surfactant anion and not enhancement that may occur for salts as reported in ref (39).

The solid red line of Figure 6a is a fit of eq 1 to the experimental data using *q*_1_ = 0.68 nC, *K*_1_ = 8.2 × 10^6^ M^–1^, *q*_2_ = 1.41 nC, and *K*_2_ = 620 M^–1^. On the other hand, the solid red line in Figure 6b is a fit of eq 1 to the experimental data using *q*_1_ = 1.12 nC, *K*_1_ = 1.5 × 10^6^ M^–1^, *q*_2_ = 2.34 nC, and *K*_2_ = 798 M^–1^. The fitted values *q*_1_ and *q*_2_ may be associated with uncertainties of approximately 0.2 nC, and taking this into account suggests that the air-bubble and dipping-probe measurements have an overlapping range of ratios of *q*_1_ and *q*_2_ (with mean values of 1.12/0.68 ≈ 1.4 and 2.34/1.41 ≈ 1.7). On the other hand, the ratios of the first quenching constants *K*_1_ differ considerably. The uncertainty of *K*_1_ obtained from the fits of eq 1 to the experimental data is of the order of 1 × 10^6^ M^–1^, and therefore cannot explain the discrepancy. We also observe that values of *K*_2_ are approximately the same for the dipping and air-bubble techniques if one accounts for an uncertainty of about 200 M^–1^, which may suggest that the difference in quenching constants occurring at small concentrations is not as significant at higher concentrations.

A puzzling observation in the dipping-probe measurements shown in Figure 6a is what appears to be an increase in charge transfer between 10 and 100 μM. This increase is not observed in the air-bubble measurements of Figure 6b. It may also appear that the dipping technique, with its larger *K*_1_, is more sensitive to changes in the SDS at small concentrations. Currently, the origin of this discrepancy is not known, but here we speculate that it could be due to the air-bubble fluctuations causing loss of sensitivity toward fine details. Although significant effort was made to couple the air bubbles to the FEP film such that close contact remained (without bouncing or other fluctuations), there were inevitable fluctuations during movement that could have reduced the ability to collect charge as the three-phase contact line passed the edge of the FEP-covered electrode. It is also possible that a thin layer of water is located between the air and the FEP surface as the air bubble moves, thus causing loss of sensitivity when using the air-bubble technique.

If one wants to explain the increase in charge transfer between 10 and 100 μM in Figure 6a, one must look beyond the two-stage charge transfer mechanism of eq 1. One possible manner to do this is by utilizing the quasi-equilibrium theory presented in ref (39). In this theory, additional charge transfer is due to the added ions removed from the electrical double layer as the three-phase contact line passes over it. The positive sodium ions are specifically contributed by the SDS molecules. Dipping the probe into aqueous solution, sodium ions in the electrical double layer beyond the shear distance x_s_ are removed from the FEP surface, thus introducing an additional charge transfer, which adds to the value *Q*_0p_. As discussed in refs^39−42^, quenching reduces water activity and needs to be accounted for through a quenching factor γ_p_ = 1/(1+*K*_qp_c), with *K*_qp_ the equilibrium quenching constant. The total charge transferred can be written as
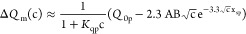
2

The area over which the charge is removed from the fluid is A, which is dependent on the size of the bubble. B is a constant depending on the surface potential as discussed in ref^39^. Assuming validity of the Gouy–Chapman theory, one may set .^39,55^ Here, *k*_B_ is Boltzmann’s constant, e the electronic charge, T the temperature, and ϕ_d_ is the inner potential of the diffusive electrical double layer where the Stern layer begins. This potential is not known, but it is seen that when  one has B ≈ −ϕ_d_. On the other hand,  results in .

Note that the theory leading to eq 2 only accounts for one quenching effect in the sense discussed in connection with eq 1, i.e., the quenching that occurs at larger concentrations. Thus, *K*_qp_ in eq 2 is expected to be of the same order of magnitude as *K*_2_ in eq 1. At smaller concentrations, eq 2 suggests that electrical double layer effects dominate, and the magnitude of the shear distance x_s_ and also to some extent the value of AB determine the charge transfer at smaller concentrations. For salts, previous works have suggested that the shear distance *x*_s_ is small (of the order of 50 nm) and there is an enhancement of charge transfer at small concentrations.^39,40^ However, surface-active molecules may form a blocking layer such that the shear distance increases and alters the charge transfer.

The dashed blue line of Figure 6a shows a fit of eq 2 to the experimental data with *Q*_0p_ = 2.0 × 10^–9^ C, AB = −3.7 × 10^–7^ Vm^2^, *K*_qp_ = 1510 M^–1^, and *x*_s_ = 127 × 10^–9^ m. Assuming *B* = −0.1 V as in ref (39), one obtains an area *A* = 3.7 × 10^–6^ m^2^, which represents the charge collection area as also discussed in ref (39). The solid line of Figure 6b shows a fit of eq 2 to the experimental data with *Q*_0p_ = 3.4 × 10^–9^ C, AB = −2.6 × 10^–7^ Vm^2^, *K*_qp_ = 1636 M^–1^, and *x*_s_ = 57 × 10^–9^ m. It is observed that eq 2 fits well to the experimental data in both Figure 6a,b and also that the peak in Figure 6a in the region 10 and 100 μM is reasonably well-resolved. Assuming once more that *B* = −0.1 V, the charge collection area of the air bubble is found to be smaller than the one for the dipping probe, which appears to be consistent with the explanation of reduced air–FEP contact due to either bubble fluctuations or a thin water layer. A notable difference in the two fits of eq 2 in Figure 6a,b is that the *x*_s_ obtained for the air bubbles is only about half that obtained for the dipping probe. Although this may suggest that the charge transfer should increase slightly at higher concentrations in the case of air bubbles, one must be careful drawing this conclusion since there is no notable peak between 10 μM and 1 mM in Figure 6b and the air-bubble measurements are probably less sensitive at smaller concentrations.

### 3.2 Charge Transfer Due to CTAB

Adding CTAB to water and recording the change in charge transfer versus time may give rise to a curve as shown in Figure 5b. As opposed to SDS, CTAB only reduces the charge transfer in the manner seen in Figure 5b, and there is no recovery of charge transfer as in Figure 5a. Figure 5b shows an example wherein 0.1 mL of 0.1 mM CTAB solution is added near the dipping probe. Since the total volume of deionized water is 70 mL, the final CTAB concentration should be about 0.14 μM CTAB. The charge transfer Δ*Q*_m_ (i.e., the peaks) is found to decrease from about 1.9 nC to about 0.6 nC. The trend-line in Figure 5b is the function Δ*Q*_m_=*Q*_a_+*Q*_b_ exp(−*t*/*t*_CTAB_), with *Q*_a_ = 0.7 nC, *Q*_b_ = 1.2 nC, and *t*_CTAB_ = 1.5 s. The monotonous decay of Δ*Q*_m_ suggests that the shear force used during dipping is not large enough to remove CTA^+^ from the surface. On the other hand, as shown in Figure 3, if one replaces the CTAB-containing solution with deionized water to do a rinse, much of the initial surface charge is recovered, which suggests that a large fraction of the surfactant is removed from the FEP surface.

**Figure 8:**
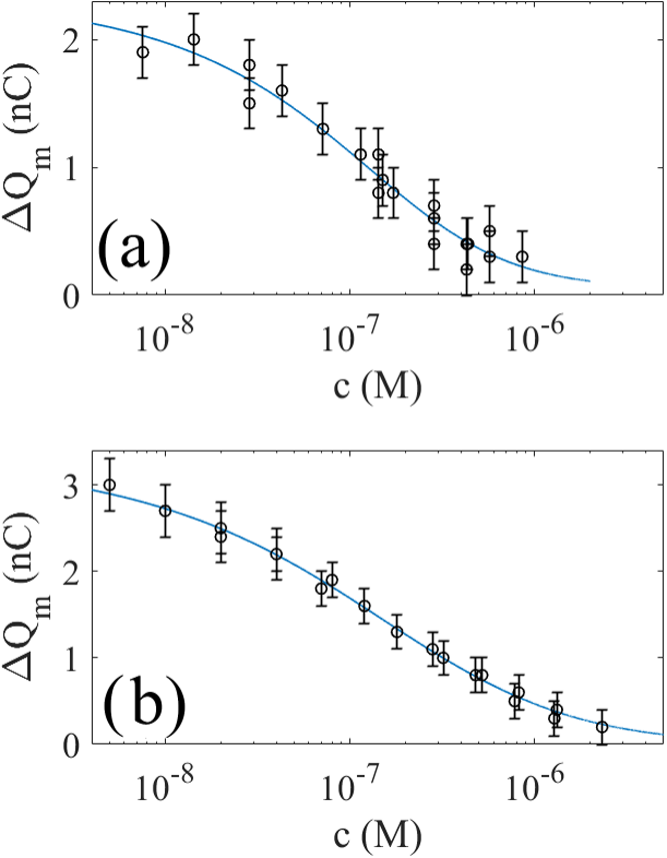
In panel (a), the circles are measured charge transfer versus CTAB concentration using the dipping probe in Figure 2a, whereas in panel (b), the circles represent the measured charge transfer using the air-bubble method in Figure 2b. The solid blue lines are fits of eq 3 to the experimental data.

In the following, the reported charge transfer reached a stable value before measurements were taken. The black circles in Figure 8a show the measured charge transfer Δ*Q*_m_ as a function of CTAB concentration using the dipping probe described in Figure 2a and Section 2.3. For pure water, the measured charge is about 2 nC, after which it decreases monotonously. The black circles in Figure 8b show the measured charge transfer using the air-bubble method described in Section 2.4. Again, it is seen that the charge transfer decays monotonously.

The data in Figure 8 can be well-explained using a single-stage quenching mechanism described in ref (41) and applied in refs^40,42^. However, it should be emphasized that the mechanism is different from that seen for SDS, as discussed in the following. In water, CTAB dissociates into CTA^+^ and Br^–^ ions. It was demonstrated in ref (39) that chloride anions play an insignificant role in altering the water activity and quenching the charge transfer. It is very likely that the same is valid also for bromide anions (Br^–^) in CTAB, and for this reason, it is believed that their contribution to the observed charge transfer quenching can be neglected. Thus, the entire charge transfer quenching is attributed to CTA^+^. Adsorption of CTA^+^ ions on the FEP surface is probably dominated by dispersion forces, except for the relatively sparse regions, where electrostatic interactions take place. With this in mind, the process may be hypothesized to take place as depicted in an artistic manner in Figure 7d–f. For small concentrations, the positive hydrophilic head of the CTA^+^ diffuses toward the interface and attach there to neutralize the negative surface charge as seen in Figure 7d. A considerable part of the hydrocarbon chain may be aligned parallel with the FEP surface,^44^ but some electrostatic interaction may also occur such that the negative charge is neutralized. Eventually, all of the negative charge has been quenched by positive hydrophilic headgroups as seen in Figure 7f, and the charge transfer is zero.

The adsorption of CTA^+^ at the FEP surface is more efficient than SDS^–^ due to its positive charge causing electrostatic interactions combined with its ability to spread over a larger surface due to dispersion forces, as depicted in Figure 7e,f. The number of negative charges (present in pure water) that have been screened by adsorbing CTA^+^ is denoted as N_s_, while the number of charges that are not screened is N*_n_*, such that the free fraction is γ_r_ = N*_n_*/(N*_n_* + N_s_) = 1/(1 + N_s_/N*_n_*). Let *q*_1_ be the magnitude of charge that can be screened, and assume that *K*_1_c = N_s_/N*_n_*, where *K*_1_ is the equilibrium quenching constant. The resulting charge transfer is then
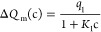
3

We note that eq 3 has the same form as eq 1, but with *q*_2_ = 0 and *K*_2_ = 0. It should be mentioned that eq 2 also reduces to eq 3 when *B* = ϕ_d_ = 0, *Q*_0p_ = *q*_1_, and *K*_qp_ = *K*_1_. That is, if CTA^+^ adsorbs near or entirely removes the negative FEP surface charge, then the inner potential of the diffusive electrical double layer where the Stern layer begins may be reduced significantly. It is also possible that the potential changes sign such that ϕ_d_ < 0 and *B* > 0, in which the second term in eq 2 reduces the charge as discussed in connection with acids in ref (39). However, since the charge transfer versus CTAB concentration is well-modeled using eq 3, it appears that the second term in eq 2 is either small such that *Q*_0p_ ≫ 2.3 AB√ce^–3.3√cx_sp_^ or that its contribution at certain values of the parameters B and *x*_s_ does not alter the curve shape significantly. Based on the available experimental data, it is not possible to state with certainty which of these two situations occur.

The solid blue line of Figure 8a is a fit of eq 1 to the experimental data using *q*_1_ = 2.29 nC and *K*_1_ = 1.1 × 10^7^ M^–1^. The solid blue line in Figure 8b is a fit of eq 1 to the experimental data using *q*_1_ = 3.07 nC and *K*_1_ = 7.7 × 10^6^ M^–1^. As with SDS, we note that the dipping charge method gives a larger quenching constant *K*_1_ than the air-bubble method, probably because there are systematic deviations due to either a thin layer of water or bubble fluctuations as discussed in the previous section.

From Figure 8, it is seen that it takes about 1 μM CTAB to neutralize a charge on the order of 2 nC on the surface of the fluoropolymer. Since 2 nC corresponds to 2 × 10^–9^ C/(10^5^ C/mol) = 2 × 10^–14^ mol of ions, and the probe collects charge in a planar region parallel to the fluoropolymer surface of about 10^–5^ m^2^,^39^ the surface concentration is expected to be of the order of 2 nM/m^2^. On the other hand, the saturated excess concentration of surfactant at the air–water interface has been estimated to be 3 μM/m^2^,^44^ which is 3 orders of magnitude larger than the surface concentration needed to neutralize the negative FEP surface charge. It should therefore be clear that the charge transfer measurements presented here are very sensitive to the CTA^+^ ions, and certainly much more sensitive than the surface tension measurements presented in Figure 1b.

In terms of volume, one could imagine that an equivalent charge came from a volume of 2 × 10^–14^ mol/10^–6^ mol/L = 2 × 10^–8^ liter (or 10^–11^ m^3^). As demonstrated in ref (39) and in the previous section, the probe collects charge in a planar area parallel to the fluoropolymer surface of at most 10^–5^ m^2^, which means that a vertical distance of at least *L* ≈ 1 μm (2 × 10^–11^ m^3^/10^–5^m^2^ = 2 × 10^–6^ m) is required to collect in all of the required CTA^+^ to entirely quench the negative charge on the fluoropolymer surface. If one assumes normal diffusion with diffusion constant *D* ≈ 10^–9^ m^2^/s, the time required for the CTA^+^ to diffuse to the polymer surface would then be τ_diff_ ≈ L^2^/*D* ≈ 4 × 10^–3^ s. This time scale is much faster than the time the probe is dipped in liquid or *t*_CTAB_ = 1.5 s from Figure 5b, thus suggesting that diffusion to the surface does not limit the charge transfer.

From Figure 1b, it can be seen that the cmc of CTAB is about 1 mM, i.e., about 3 orders of magnitude larger than the concentration at which the measured charge transfer becomes negligible. As with SDS, it is believed that the formation of micelles does not play a role in charge transfer, but a study of this at higher concentrations is outside the scope of the current work.

### 3.3 Charge Transfer Due to Triton X-100

**Figure 9:**
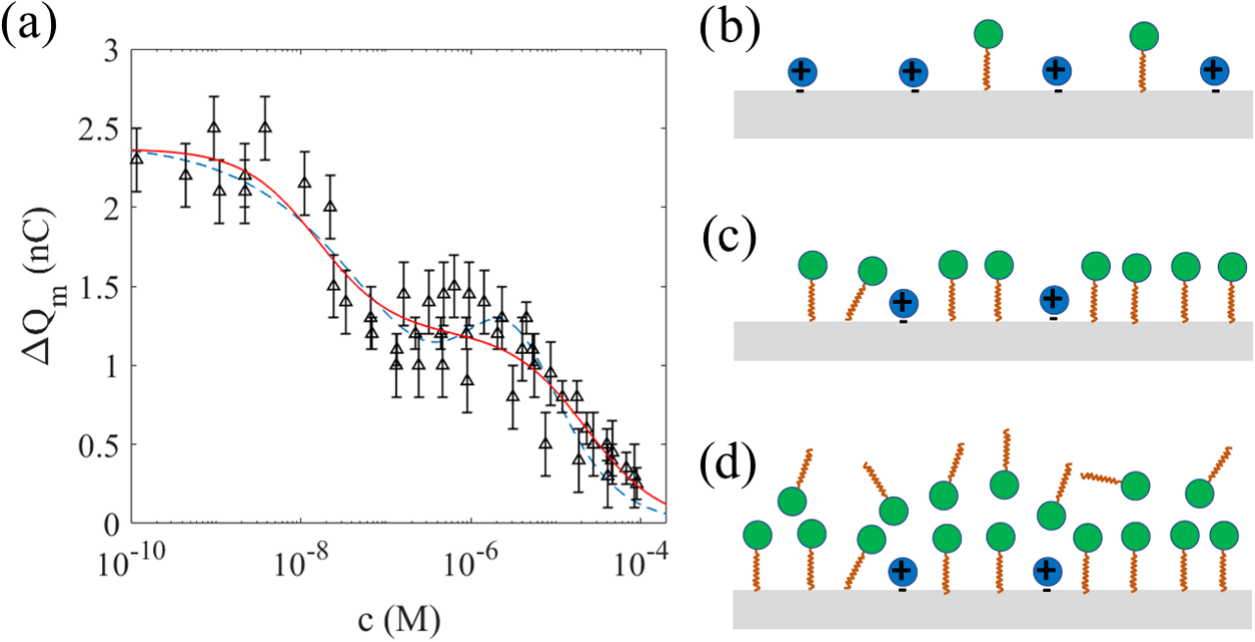
In panel (a), the triangles are the measured charge transfer versus Triton X-100 concentration using the dipping probe in Figure 2a. The red solid line is a fit of eq 1, and the blue dashed line is a fit of eq 2, to the experimental data. In panels (b)–(d), an artistic and schematic drawing of the charge transfer quenching by Triton X-100 is shown at small concentrations (b), at the end of the first quenching stage (c), and at the end of the second quenching stage (d).

The black triangles in Figure 9a show the measured charge transfer Δ*Q*_m_ as a function of Triton X-100 concentration using the dipping probe described in Figure 2a and Section 2.3. The charge transfer is found to behave in a qualitatively similar way as SDS. That is, the charge transfer decreases from about 2 to 1 nC as the concentration increases to 0.1 μM Triton X-100 but does not decrease further after that until about 5 μM. The measurements cannot resolve whether there is a charge peak between 0.1 and 5 μM since the uncertainty between trials is too large. In some trials, there appears to be a small peak, in others not, but the origin of these variations could not be determined. After 5 μM, the charge transfer decreases monotonously to zero at about 0.1 mM, which coincides with the cmc of Triton X-100 according to Figure 1b. Measurements above cmc are outside the scope of the current work, but as with SDS it is believed that the micelles form in the bulk and not at the FEP surface. It is likely that the formation of micelles does not alter the charge transfer.

The two-stage charge quenching observed in Figure 9a suggests that eq 1 can be used to explain the experimental observations. However, it is clear that the quenching mechanisms cannot be entirely the same as for SDS since Triton X-100 is not charged. On the other hand, it should also be observed that it is very likely that the hydrophobic tail arranges near the FEP surface, as shown in Figure 9b, thus disrupting the hydrogen bonding network and reducing the water activity, just as in the case of SDS. As the concentration of Triton X-100 is increased, some of the surface protons and negative surface charge are removed by the Triton X-100 molecules, while some of the more resilient charge sites remain. When enough Triton X-100 molecules are present at the FEP surface, a patchy monolayer is formed surrounding the resilient negative charges, and the first quenching stage is complete, as shown in Figure 9c. Further increase in concentration does not change the measured charge transfer in any significant manner, since most of the Triton X-100 molecules coming in from the bulk locate in the vicinity of the hydrophobic heads a distance away from the FEP surface. Thus, the charge transfer remains nearly constant between 0.1 and 5 μM. However, eventually, the concentration of Triton X-100 molecules becomes large enough to start surrounding the surface protons and the negative surface charge closest to the FEP surface. This is illustrated in Figure 9d. It is hypothesized that when the three-phase contact line moves over the FEP surface, the Triton X-100 molecules shield the surface protons from being removed from the FEP surface. This hypothesized shielding mechanism is similar to that proposed for SDS.

The perhaps simplest mathematical model that can take into account the main factors discussed above is eq 1, and a derivation thereof follows the same lines as for SDS. The solid red line of Figure 9a is a fit of eq 1 to the experimental data using *q*_1_ = 1.17 nC, *K*_1_ = 6.30 × 10^7^ M^–1^, *q*_2_ = 1.20 nC and *K*_2_ = 4.44 × 10^4^ M^–1^. Table 1 provides a comparison of the fitting constants, which allows one to compare the results for different surfactants.

**Table 1:** Fitting Parameters Obtained When Fitting eqs 1 and 2 to the Experimental Data Using the Dipping Probe

species	model eq 1				model eq 2		
	*q*_1_ (nC)	*K*_1_ (M^–1^)	*q*_2_ (nC)	*K*_2_ (M^–1^)	*K*_qp_ (M^–1^)	AB (nVm^2^)	*x*_s_ (nm)
SDS	0.68	8.2 × 10^6^	1.41	620	1510	–370	127
CTAB	2.29	1.1 × 10^7^	0		1.1 × 10^7^	0	
triton	1.17	6.3 × 10^7^	1.20	4.44 × 10^4^	1.1 × 10^5^	–2610	560
PvOH	2.6	8.5 × 10^9^	0		8.5 × 10^9^	0	

It is noted that *q*_1_ is nearly twice as large for Triton X-100 (*q*_1_ = 1.17 nC) compared to SDS (*q*_1_ = 0.68 nC), which may suggest that Triton X-100 provides a stronger initial screening than SDS at low concentrations during the first stage of quenching. This is not unexpected since Triton X-100 is nonionic and the hydrophobic tails can therefore populate the FEP surface more densely without the hydrophilic heads experiencing an electrostatic repulsion from the already existing negative charge on the FEP surface. When the negative charge on the FEP surface is more effectively surrounded by surfactant molecules, the charge transfer might be more strongly quenched. That is, the first stage of the charge quenching is more effective and therefore gives rise to a larger *q*_1_ for Triton X-100. Here, *K*_1_ is larger for Triton than for SDS, which shows that the saturation of the first quenching stage is reached at smaller molar concentrations. At the same time, it should be noted that unlike SDS, Triton X-100 provides no free cations which may enhance the charge transfer. During the second stage of quenching, the value of *K*_2_ is much smaller for SDS (*K*_2_ = 620 M^–1^) than for Triton X-100 (*K*_2_ = 4.44 × 10^4^ M^–1^), possibly also related to the difference in electrostatic repulsion, which allows the Triton X-100 molecules to more effectively screen the charges of the electrical double layer, which otherwise participate in charge transfer.

It should be pointed out that it is also possible to model the experimental data in Figure 8a using eq 2. In Figure 9a, the dashed blue line shows a fit of eq 2 to the experimental data with *Q*_0p_ = 2.4 × 10^–9^ C, AB = −2.61 × 10^–6^ Vm^2^, *K*_qp_ = 1.9 × 10^5^ M^–1^, and *x*_s_ = 560 × 10^–9^ m. Perhaps most notable is that AB and *x*_s_ are much larger than the same values for SDS. The value of *x*_s_ is about four times larger for Triton X-100, and the theory behind eq 2 then suggests that the Triton X-100 molecules extend the shear thickness at which the moving three-phase contact line may sweep away charge. This is consistent with Figure 9c,d, where the Triton X-100 molecules essentially shield the inner part of the electrical double layer such that only positive charges positioned beyond *x*_s_ can be removed by the three-phase contact line. The nonionic surfactant does not interact much with the charges in the electrical double layer, but rather provide hindrance and a buffer such that the electrical double layer becomes more dilute and extends farther out from the FEP surface.

In ref (56), it was demonstrated that zwitterions do not influence the electrostatic forces in the electrical double layer and potentials between some negatively charged surfaces like mica–mica and silica–silica. One might naively expect that this would also be the case for a nonionic surfactant like Triton X-100 and that this would prevent any significant influence of this surfactant on the electrical double layer except as a spacer increasing the shear length as suggested above. It must be emphasized that although ions further out in the diffuse part of the electrical double layer are not shown in Figure 9d, the theory of eq 2 presented in ref (39) relies on the removal of positive ions in the electrical double layer beyond a distance *x*_s_ from the FEP surface. The large value of AB found for Triton X-100 could be due to these positive charges in the diffuse part of the electrical double layer being positioned further apart in an electric field due to the surfactant spacer, thus giving rise to a value of the voltage-dependent parameter B larger than that for SDS. However, given the uncertainty between trials and the deviations of eq 2 from the experimental data, one should be careful in extending this interpretation too far.

### 3.4 Charge Transfer Due to PvOH

While fluoropolymer surfaces often are considered inert on which adsorption is difficult, it is known that some proteins and polymers may adsorb to these hydrophobic surfaces under certain environmental conditions, probably due to hydrophobic interactions with a reduction of interfacial energy as water molecules are displaced from the interface.^47^ Two reports have demonstrated that PvOH stands out in its ability to alter the surface of FEP by forming a thin film of the order of 10 Å, which increases the polarity of the surface.^48,49^ For this reason, it was decided to measure the influence of this particular polymer in water on charge transfer as a reference for the surfactants studied.

**Figure 10:**
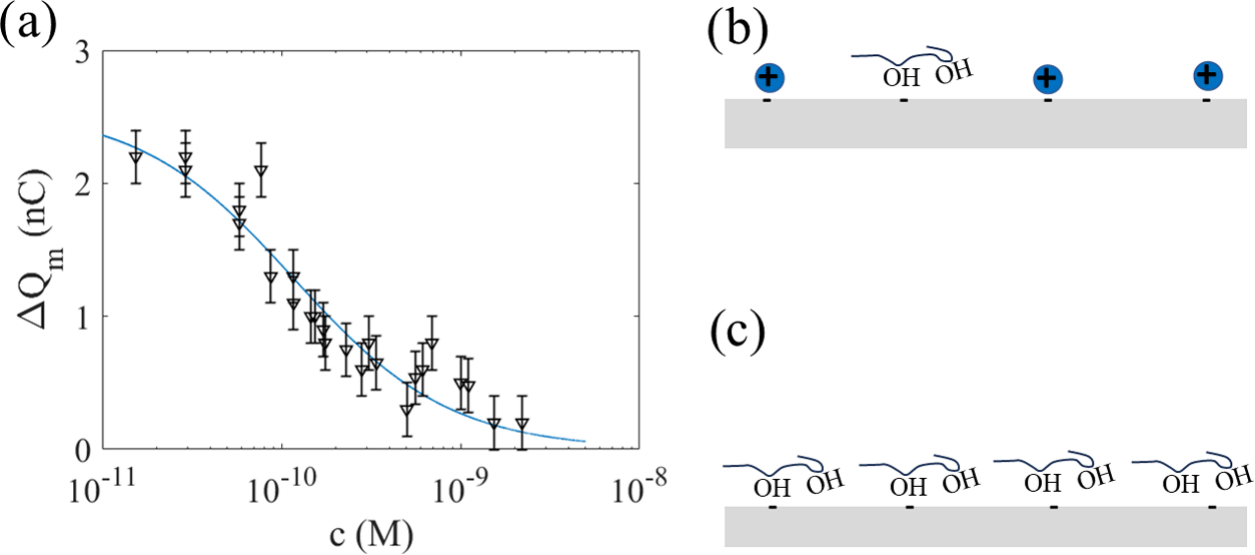
In panel (a), the triangles are the measured charge transfer versus PvOH molar concentration. The blue dashed line is a fit of eq 3 to the experimental data. In panels (b) and (c), a schematic and artistic view of how the PvOH molecules displace the water and reduce water activity near the FEP surface as the polymer concentration increases is shown.

The black triangles in Figure 10a show the measured charge transfer Δ*Q*_m_ as a function of PvOH concentration using the dipping probe described in Figure 2a and Section 2.3. Here, the concentration in M was found assuming the given mean molecular weight of 1,30,000 g/mol from the vendor. The charge transfer decreases and becomes unmeasurable with the dipping-probe technique much above 1 nM.

The simplest model for the interpretation of the experimental data is a model exhibiting a quenching mechanism as discussed for CTAB. However, while the positive charge of CTA^+^ combined with the dispersion forces causing the surfactant anion to align with the FEP surface could explain the quenching caused by CTAB, the quenching caused by PvOH is of a different origin since there is no obvious positive charge present. Instead, it is likely that the adsorption of PvOH is due to water displacement and hydrogen bonding network disruption.^47^ When PvOH is introduced into water and the molecules adsorb at the FEP surface, the local hydrogen bonding network is disrupted when water molecules are displaced from the FEP surface. An artistic view of this is shown in Figure 10b,c. The water activity decreases, the electrical double layer transfers less charge, and the subsequent charge transfer diminishes. It is also known that the surface becomes significantly more hydrophilic and that the wetting properties therefore change.^47−49^ Here, we will not consider these changes in wetting properties, and instead assume similar steps as in deriving eq 3. That is, we assume that the number of negative charges (present in pure water) that have been removed by adsorbing PvOH molecules is denoted as *N*_r_, while the number of charges that remain is *N*_e_, such that the fraction is γ_r_ = *N*_e_/(*N*_e_ + *N*_r_) = 1/(1 + *N*_r_/*N*_e_). The resulting charge transfer is then given in eq 3.

The blue line in Figure 10a corresponds to a fit of eq 1 with *q*_1_ = 2.6 nC and *K*_1_ = 8.5 × 10^9^ M^–1^. The value of *K*_1_ is very high due to the particular affinity of PvOH molecules toward the hydrophobic surface. In the low-concentration regime, single molecules adsorb to the FEP surface by orienting their OH groups in such a way that the entire molecule displaces water molecules and participates in the hydrogen bonding structure to align with the solid surface; see Figure 10b. At higher concentrations, the hydrogen bonding is expected to further stabilize and entangle the PvOH molecules to form a partially crystalline film that is at least 10 Å thick that makes the entire surface hydrophilic.^47−49^ This is depicted in Figure 10c. These partially crystalline films also make it very hard to just rinse the film away as demonstrated in Figure 3b.

While the adsorption mechanism for PvOH is different from CTAB, Figure 10a clearly demonstrates that the charge transfer can be modeled in the same way using eq 3. Furthermore, also the charge transfer versus concentration of alcohols like methanol and glycerol in water can be understood using the same model,^40^ which further suggests that this model is rather universal when there is a single-stage mechanism at play.

## 4 Conclusions

In this study, the influence of different surfactants on charge transfer is studied as a hydrophobic polymer surface is dipped into a solution, or an air bubble is passed over the same type of hydrophobic surface. In contact with pure water, the surface acquires a net negative. It is found that cationic surfactants effectively quench the negative charges in a single stage, whereas nonionic and anionic surfactants exhibit a more complicated charge transfer response.

One way to interpret the results for nonionic and cationic surfactants is to consider two independent quenching stages. The first stage suggests formation of a patchy layer of surfactant molecules that position their hydrophobic tails near the hydrophobic surface. The interaction with the negative charge on the polymer surface depends on whether the surfactant is anionic or nonionic. In either case, one observes partial quenching of the existing negative surface. However, when there is no more room for hydrophobic tails near the polymer surface, the charge cannot be quenched further by this mechanism. The nonionic surfactant Triton X-100 was found to screen more of the negative charge at lower concentrations than the anionic surfactant SDS in this first stage. This could be due to the reduced electrostatic repulsion between the poly(ethylene oxide) hydrophilic head groups of Triton X-100 and the negative charge on the FEP surface, thus allowing also the hydrophobic group to populate the FEP surface more densely and thereby screening more of the charge transfer as the three-phase contact line passes by. In the second quenching stage, complete quenching is reached when the surfactant surrenders and physically screens the positive surface charge from being removed by the three-phase contact line.

While the one- and two-stage quenching mechanisms appear to explain most of the features observed for all of the surfactants studied, it must be emphasized that there are some deviations. In particular, it is observed that in particular, SDS (and to a smaller degree Triton X-100) gives rise to a small increase in charge transfer after the first quenching stage. In ref (39), such increases in charge transfer were explained as due to removal of additional positive counterions from the diffuse part of the electrical double layer. If this assumption is made also here, it is found that the theory of ref (39) can explain the charge transfer quenching as well as the small increase in charge transfer by requiring that Triton X-100 has a much larger shear thickness for removal of ions than SDS. A possible explanation for this may be that the nonionic surfactant molecules do not interact so strongly with the charges in the electrical double layer but rather form a layer that extends the thickness of the electrical double layer or allow ions further away from the FEP surface to be swept away as the three-phase contact line passes by.

Charge transfer is of crucial importance for energy harvesting devices based on water waves, water droplets in air, or air bubbles in water passing over hydrophobic surfaces. In the current work, it has been demonstrated that very small amounts of surfactants may reduce the charge transfer when dipping probes into water or letting air bubbles move past them. In the case of CTAB, concentrations of a few μM are required to quench the charge transfer, whereas for SDS one needs concentrations about thousand times larger. In any case, the concentrations required to significantly alter the charge transfer do not give rise to significant changes in surface tension at the air–water interface and are therefore difficult to monitor simply using tensiometers often used to monitor surfactants. In energy harvesting, one is interested in understanding how the harvesting device works under normal conditions in tap water, seawater, or rainwater. In most of these situations, surfactants are not present, but in some situations, the water might be contaminated, and the current study demonstrates that one needs to ensure that this has been accounted for when testing the device and that tensiometers often used to monitor surfactants are not adequate for this task. The cleanliness of the hydrophobic surface itself is also of importance, and proper cleaning in surfactant-free solutions is needed before testing commences. Contaminated cleaning chemicals may give rise to a layer of surfactant that is not easily rinsed with pure water. In addition to paying attention to cleaning procedures, one should also be aware of the influence of surfactants if they are needed in, for example, monitoring of liquids. The fact that similar one- and two-stage charge transfer reduction behavior was observed using two different techniques, dipping or air bubbles, suggests that one should take this into account both when studying the fundamental mechanisms for charge transfer as well as in applications.
